# The Prime Time for Flexible Ureteroscopy for Large Renal Stones Is Coming: Is Percutaneous Nephrolithotomy No Longer Needed?

**DOI:** 10.5152/tud.2023.23142

**Published:** 2023-09-01

**Authors:** Senol Tonyali, Hakan Bahadir Haberal, Francesco Esperto, Zeeshan Hamid, Lazaros Tzelves, Amelia Pietropaolo, Esteban Emiliani

**Affiliations:** 1 Department of Urology, Istanbul University Istanbul School of Medicine, Istanbul, Turkey; 2 European Association of Urology, Young Academic Urologist, Urolithiasis and Endourology Working Group, Arnhem, Netherlands; 3 Department of Urology, University of Health Sciences, Ankara Sanatorium Training and Research hospital, Ankara, Turkey; 4 Department of Urology, Campus Bio-Medico, University of Rome, Rome, Italy; 5 Department of Urology, Father Muller Medical College, Mangalore, India.; 6 Department of Urology/Urooncology, University College of London Hospitals (UCLH), London, United Kingdom; 7 Department of Urology, University Hospital Southampton NHS Trust, Southampton, United Kingdom; 8 Department of Urology, Fundacio Puigvert, Autonomous University of Barcelona, Spain

**Keywords:** Urolithiasis, nephrolithotomy, retrograde intrarenal surgery, holmium laser, thulium laser

## Abstract

Advances in laser technology and surgical telescopic systems have propelled retrograde intrarenal surgery (RIRS) to the forefront as a viable alternative to percutaneous nephrolithotomy (PCNL). Currently, RIRS is being increasingly utilized as a treatment option, even for kidney stones larger than 2 cm. In this narrative review, we aimed to take a snapshot of current practice in renal stone treatment and the latest technological and technical developments and to evaluate the efficacy of RIRS in larger renal stones. With low complication rates and acceptable stone-free rates, RIRS offers patients a less invasive option with favorable outcomes. There are insufficient data comparing PCNL with RIRS using a new-generation high-power laser and suctioning ureteral access sheath (UAS). Further studies with novel lasers and UAS could provide superiority in terms of RIRS. It is crucial to take into account various patient-specific considerations, such as stone location and burden, when deciding on the appropriate treatment approach.

**Main Points**

- The ablation efficacy (mm3/second), the ablation efficiency (mm3/Joule), and the laser activity (ratio between the laser active time and the lithotripsy time) are three different parameters to evaluate the Ho:YAG lithotripsy performance.
- In mid 2010’s suctioning UAS has been proposed to decrease intrarenal pressure and improve surgical visualization.
- Recently, a flexible vacuum-assisted ureteral access sheath (FV-UAS) was introduced to cross the ureteropelvic junction into the renal pelvis and caliceal system.
- There is insufficient data comparing PCNL with RIRS using a new generation high-power laser and suctioning ureteral access sheath.

## Introduction

Urinary stone disease is a frequent urological disorder, with an estimated prevalence varying between 1% and 15%. There has been a steady increase in the prevalence of urolithiasis during the past decades.^1^ Given the lack of an effective medical treatment for the majority of urinary calculi, surgical interventions remain the mainstay of treatment for patients with symptomatic stones. Although open lithotomy was the main surgical method in the past, thanks to technological advancements, endourologic interventions, such as flexible ureteroscopy and percutaneous nephrolithotomy (PCNL), have become the standard treatments for urolithiasis in more recent years.^2^

Advances in laser technology and surgical telescopic systems have propelled retrograde intrarenal surgery (RIRS) to the forefront as a viable alternative to PCNL. Currently, RIRS is being increasingly utilized as a treatment option, even for kidney stones larger than 2 cm. As a consequence of this, there has been a rise in the number of studies comparing the outcomes of RIRS and PCNL in the literature.

In this narrative review, we aimed to take a snapshot of current practice in renal stone treatment and the latest technological and technical developments and to evaluate the efficacy of RIRS in larger renal stones.

### Literature Review

#### Laser Technology Advancements

The holmium : yttrium–aluminum–garnet (Ho:YAG) laser has generally been the most utilized laser for lithotripsy during the last 2 decades. Thanks to the technological improvements, nowadays, several highly effective and powerful lasers are available in the market. The Ho:YAG laser operates by generating light through electricity that stimulates a solid-state active medium. The emission of photons out of protons is stimulated by electricity at an infrared wavelength of 2140 nm, as they return to their resting state. The power of lases varies between 10 and 140 W, depending on the manufacturer. Lasers with a power higher than 35 W are classified as high-power lasers, while lasers with a power less than 35 W are low-power lasers.^3^

The ablation efficacy (mm^3^/s), the ablation efficiency (mm^3^/joule), and the laser activity (ratio between the laser active time and the lithotripsy time) are 3 different parameters that used to evaluate the Ho:YAG lithotripsy performance. The volume of stone dust or fragments created per unit of time is called ablation efficacy, and it is related to the pulse energy, the pulse frequency, and the pulse length. The main technical difference between a high-power laser and a low-power laser is precisely the pulse frequency. If every pulse is effectively delivered to the stone surface, then a higher pulse frequency increases the ablation efficacy. Some researchers reported that there is not sufficient in vivo data to support the superiority of a high-power Ho:YAG laser compared to a low-power laser regarding ablation efficacy. One of the main limitations suggested was poor visibility because of the dust, leading to repeated interruptions of the procedure.^4^

In a systematic review and meta-analysis comparing the ureteroscopic lithotripsy performance of low-power and high-power lasers, Ventimiglia et al reported that the operative time with high-power lasers was significantly shorter than that with low-power lasers. However, stone burden was 2-fold higher in low-power laser studies. The stone-free rate (SFR) (81% vs. 82%, *P* > .05) and complication rates (*P* = .12) were comparable between the 2 laser settings. The authors concluded that the faster operative time, particularly observed in laboratory studies, might not be applicable to clinical practice. They suggested at least 3 intervening factors that may derange the favorable outcomes observed in the laboratory, namely, quality of vision, caliceal distensibility, and microbleedings.^5^ In a recent study by Pietropaolo et al,^6^ the authors reported their ureteroscopic laser lithotripsy outcomes for renal stones > 15 mm using a high-power Ho:Yag laser. The final SFR was 94%, with a complication rate of 3.9%. The authors concluded that ureteroscopic laser lithotripsy utilizing dusting and pop-dusting techniques are successful and safe in the treatment of large, bilateral, or multiple renal stones with high SFR and low complication rates.

New pulse modulation (PM) technologies are also available for Ho:YAG lasers. In a systematic review comparing the lithotripsy performance of lasers with different PMs, researchers found a statistically significant difference in terms of operative time in 6 out of 7 studies favoring new-generation PM technologies. Five out of 6 studies favor new-generation PM technologies in terms of fragmentation time. Both of the 2 studies evaluating retropulsion found new-generation PM superior to regular PM. However, no pooled differences were detected regarding SFR, lasing, and total operative time, and complication rate between Moses technology and regular PM.^7^

### Comparative Studies Between Retrograde Intrarenal Surgery and Percutaneous Nephrolithotomy

With the advancements in laser technology, devices with distinct properties are being utilized in current medical practice. Consequently, studies investigating the efficacy of these lasers have begun to emerge in the scientific literature. The thulium fiber laser has gained increased attention in the last few years. Perri et al^8^ conducted a randomized prospective study comparing the outcomes of mini-PCNL (mPCNL) and RIRS using the SuperPulsed thulium fiber laser for the treatment of 10 mm and 20 mm kidney stones. Their research findings indicated that the overall SFRs were comparable between the 2 procedures. However, they observed that RIRS had a statistically significant higher SFR specifically for upper calyceal stones. These findings suggest that RIRS using the SuperPulsed thulium fiber laser may offer an advantage over mPCNL, specifically for upper calyceal stones. Similarly, Taratkin et al^9^ conducted a study wherein they prospectively enrolled patients with kidney stones measuring 2 cm and larger, evaluating the efficacy of RIRS. Their research, utilizing the SuperPulsed thulium fiber laser, demonstrated that RIRS yields comparable success rates to PCNL while also exhibiting similar complication rates. 

The study conducted by Lv et al^10^ compared the outcomes of RIRS and mPCNL in patients with kidney stones >2 cm. Through propensity score matching, they aimed to create comparable groups for a more reliable comparison. The study indicated that RIRS and mPCNL achieved similar SFRs, suggesting that both procedures were effective in treating kidney stones of this size. Furthermore, the study also demonstrated certain advantages of RIRS over mPCNL in terms of hospital stay and complication rates. Similarly, Fayad et al^11^ conducted a comprehensive randomized study comparing the effectiveness of RIRS and PCNL in patients with renal pelvis stones >2 cm. According to their study, both RIRS and PCNL yielded comparable SFRs. Moreover, they demonstrated a distinct advantage of RIRS by providing a reduced risk of bleeding complications, thereby minimizing the likelihood of transfusion requirements.

The primary area where RIRS falls behind PCNL is the lower pole stone treatment. Numerous randomized controlled studies have consistently demonstrated that PCNL yields a significantly higher SFR compared to RIRS when it comes to managing lower pole stones.^12-14^ Huang et al^15^ conducted a study focusing on treatment selection for patients with lower pole stones sized between 1 and 2 cm. The research findings revealed that several factors play a crucial role in determining the appropriate treatment approach, including stone number, stone diameter, infundibulopelvic angle, infundibular length, and infundibular width. These parameters are important considerations when making decisions between RIRS and PCNL about the most suitable choice of treatment for renal stones located at the lower pole. Despite PCNL having an advantage over RIRS in managing lower pole stones, it is worth noting that meta-analyses have indicated comparable SFRs between RIRS and PCNL, specifically in the context of 2-3 cm kidney stones.^16^ These analyses have demonstrated that RIRS can achieve similar outcomes to PCNL in terms of success while also exhibiting low rates of intraoperative complications. 

Zhao et al^17^ conducted an evaluation of the outcomes of RIRS specifically in patients with kidney stones ranging from 2 to 3 cm in size. Their study findings indicated that the success rates of RIRS were relatively lower in patients with certain characteristics, including lower calyx involvement, involvement of multiple calyces, and severe hydronephrosis. Consequently, the authors suggested that prioritizing PCNL might be more appropriate for patients presenting with these features.

In a retrospective study conducted more than 10 years ago, researchers compared the surgical outcomes of RIRS and PNL for renal stones sized between 2 and 4 cm. The holmium laser was set at an energy of 1.0-1.5 J and a rate of 8-10 Hz in RIRS. A ureteral access sheath (UAS) was used in selected cases. They found that the SFR was 73.5% for RIRS and 91.2% for PCNL after a single session (*P* = .05). The SFR increased to 88.2% in the RIRS group after the second procedure.^18^

In another multi-institutional study performed in 2010, researchers presented their results of RIRS in renal stones sized 2-3 cm. The absolute SFR was 47% in this study.^19^

In a study evaluating planned staged RIRS in renal stones >2.5 cm, the authors reported that the mean number of total procedures was 1.82 and the overall SFR was 90.0%. The SFR was defined as having fragments smaller than 2 mm postoperatively.^20^ In another study with a relatively small number of patients, the overall SFR was 90%. All stones were larger than 2 cm, and the mean stone size was 3.1 cm. Patients having residual fragments smaller than 4 mm were considered stone free in that study.^21^

Patients with solitary kidneys represent an important subgroup when it comes to managing stone disease. Ensuring a high SFR while minimizing complications is crucial in this patient population. Addressing this aspect, Jiang et al^22^ conducted a meta-analysis study encompassing various studies that compared RIRS and PCNL in patients with solitary kidneys and renal calculus > 2 cm. The results of their comprehensive analysis revealed that initially PCNL demonstrated a superior SFR; however, the final SFR was found to be similar in both the RIRS and PCNL groups. In addition to this, the study showed that RIRS exhibited a low blood transfusion rate, emphasizing its safety profile and suggesting a reduced risk of bleeding complications. Importantly, the overall complication rates were similar between RIRS and PCNL, indicating that RIRS can be a viable alternative for patients with solitary kidneys who have kidney stones larger than 2 cm (Table 1).

### Ureteral Access Sheath Use in Retrograde Intrarenal Surgery

It has been shown that the use of UAS during RIRS decreases the intrarenal pressure and might help prevent infectious complications. Ureteral access sheath use also allows multiple entries and re-entries to the collecting system to extract stone fragments. However, the diameter of the UAS is still a challenging problem. The increased diameter of the UAS significantly decreases the intrarenal pelvic pressure due to the increased space between the scope and the UAS. But the use of UAS is not free of complications. The insertion of UAS might lead to serious ureteral wall injuries, and in rare cases, the distension of the ureter by UAS may decrease the ureteral blood flow and cause ureteral ischemia and stricture.^23^

In mid-2010’s suctioning, UAS has been proposed to decrease intrarenal pressure and improve surgical visualization. In a retrospective study comparing the outcomes of traditional and suctioning UAS during flexible Ureterorenoscopy (URS) for renal stones, the researchers reported that overall complications were significantly higher and the operative time was longer in the traditional URS group compared to the suctioning URS group, 24.8% vs. 11.5% (*P* < .001) and 57.0 ± 14.0 min and 49.7 ± 16.3 min, respectively (*P* < .001).^24^

In another study including 278 patients that evaluated the safety and efficacy of suctioning flexible ureteroscopy with intelligent pressure control (SFUI) in the treatment of upper urinary tract stones, the single-session SFR was 80.65% and the 1-month SFR was 82.26%. The researchers concluded that patients with stones <40 mm or Guy’s stone score of grade I are likely to be stone free after SFUI treatment.^25^

Recently, a flexible vacuum-assisted ureteral access sheath (FV-UAS) was introduced to cross the ureteropelvic junction into the renal pelvis and caliceal system. In a porcine kidney model, it has been shown that intrarenal pelvic pressures can be maintained lower than 10 cm H_2_O by increasing the negative value at any irrigation fluid velocity. They also concluded that FV-UAS can achieve complete stone-free status in RIRS by being close to the stone. However, SFR at postoperative first month was comparable between the traditional UAS group (82.9%) and the suctioning UAS group (88.8%) (*P* = .13).^26^

In a review article evaluating suctioning techniques during flexible ureteroscopy, including 12 studies, the authors revealed several benefits of different suction modalities utilized in URS in experimental and clinical studies, including increased SFR, improved intraoperative visibility, decreased operation time, and fewer complications. However, robust clinical data supportive of the routine use of suction during URS are still lacking.^27^

In a study comparing RIRS with vacuum assisted UAS (V-UAS) and mPCNL in renal stones sized between 2 and 4 cm, the authors found that operative time was significantly higher and postoperative pain was significantly less in the RIRS group compared to the mPCNL group: 72.4 ± 21.3 minutes vs. 67.4 ± 25 minutes (*P* = .042). Although the initial SFR was significantly higher in the mPCNL group (73.2% vs. 50%, *P* = .035), the final SFR was comparable between the RIRS and mPCNL groups (89.3% vs. 92.9%, *P* = .681).^28^

## Conclusion

Based on current studies, RIRS has emerged as a promising alternative to PCNL for the management of kidney stones >2 cm. With low complication rates and acceptable SFRs, RIRS offers patients a less invasive option with favorable outcomes. There are insufficient data comparing PCNL with RIRS using a new-generation high-power laser and suctioning UAS. Further studies with novel lasers and UAS could provide superiority in terms of RIRS. It is crucial to take into account various patient-specific considerations, such as stone location and burden, when deciding on the appropriate treatment approach.

## Figures and Tables

**Table 1.** Studies Comparing PCNL and RIRS

Study	Study Type	Study Population	Stone Location	Stone Size	Stone-Free Rate	Complication Rate
Perri et al (2022)	Prospective	RIRS (n = 90)	Single renal stone	10-20 mm	RIRS: 73.3%, mini-PCNL: 84.4%. For upper calyceal stones RIRS: 90.4% and for lower calyceal stones mini-PCNL: 91.6%	5.5%
		Mini-PCNL (n = 96)				5.2%
Taratkin et al (2021)	Retrospective	RIRS with SuperPulsed thulium fiber laser (n = 14)	Single renal stone	≥20 mm	85.7%	14.2%
		PCNL (n = 56)			89.3%	10.9%
Lv et al (2022)	Retrospective	RIRS (n = 81)	All kidney stone patients with or without ureteral stones	>20 mm	SFR RIRS: 74.1% mini-PCNL: 77.8%. SFR after 3 months RIRS: 97.5% mini-PCNL: 96.3%.	11.1%
		Mini PCNL (n = 81)				30.9%
Fayad et al (2022)	Prospective	RIRS (n = 61)	Single renal pelvic stone	>20 mm	72.1%	39.3%
		PCNL (n = 60)			78.3%	48.3%
Liu et al (2022)	Prospective	RIRS (n = 60)	Lower pole renal calculi in obese patients	20-30 mm	61.4%	7%
		Mini-PCNL (n = 60)			86.2%	22.4%
Huang et al (2022)	Retrospective	RIRS (n = 152)	Lower pole stone	10-20 mm	78.3	8.6%
		PCNL (n = 137)			89.1	18.2%
Zhao et al (2020)	Retrospective	RIRS (n = 147)	All patients with kidney stones	20-30 mm	66%	12.2%
		Mini-PCNL (n = 129)			93.3%	8.5%
Akman et al (2012)	Retrospective	RIRS (n = 34)	All patients with kidney stones	20-40 mm	73.5%	11.7%
		PCNL (n = 34)			91.2%	14.7%

PCNL, percutaneous nephrolithotomy; RIRS, retrograde intrarenal surgery; SFR, stone-free rate.
